# Draft Genome Sequence of “*Candidatus* Cronobacter colletis” NCTC 14934^T^, a New Species in the Genus *Cronobacter*

**DOI:** 10.1128/genomeA.00585-14

**Published:** 2014-06-19

**Authors:** Naqash Masood, Emily Jackson, Karen Moore, Audrey Farbos, Konrad Paszkiewicz, Ben Dickins, Alan McNally, Stephen Forsythe

**Affiliations:** a Pathogen Research Group, School of Science and Technology, Nottingham Trent University, Nottingham, United Kingdom; b Wellcome Trust Biomedical Informatics Hub, Biosciences, University of Exeter, Exeter, United Kingdom

## Abstract

Members of the *Cronobacter* genus are associated with serious infections in neonates. This is the first report of the draft genome sequence for the newly proposed species *Cronobacter colletis*.

## GENOME ANNOUNCEMENT

Due to the association of *Cronobacter* with fatal neonatal infections, there is an international requirement for powdered infant formula to be microbiologically tested for all members of the *Cronobacter* genus (1). “*Candidatus* Cronobacter colletis” is a previously undescribed species closely related to *Cronobacter zurichensis*. Therefore, genome sequencing and public access of this newly described species are warranted for a better understanding of the diversity of the genus and improved detection methodology. This was undertaken using the type strain “*Candidatus* Cronobacter colletis” NCTC 14934.

Bacterial DNA was extracted from 1-day cultures using a GenElute bacterial genome kit (Sigma-Aldrich) and sequenced using an Illumina HiSeq 2500 sequencing platform. A total of 1,501,270 high-quality paired-end reads were generated, with 16-fold coverage. *De novo* assembly was performed using Velvet (version 1.1.09) (2). The genome was distributed in 42 contigs, with a total size of 4,261,112 bp and a G+C content of 57.07%.

A phylogenetic tree based on seven housekeeping genes (3,036 bp concatenated length) from *Cronobacter* BIGSdb (http://www.pubMLST.org/cronobacter; http://dx.doi.org/10.6084/m9.figshare.1032771) suggests that “*Candidatus* Cronobacter colletis” is a member of the *Cronobacter* genus, with a near match to *C. zurichensis*. A comparison of the “*Candidatus* Cronobacter colletis” genome with the genome sequence available for *C. zurichensis* LMG 23730^T^ (3) revealed an average nucleotide identity (ANI) of 87.18%. A formal description of “*Candidatus* Cronobacter colletis” is currently in progress.

For further annotation, we used the SEED-based automated annotation system provided by the RAST server (4), which identified 3,967 coding sequences (CDSs) and 109 RNAs. The CDSs included those for copper homeostasis, iron acquisition, multidrug efflux pumps, arsenic, cobalt, zinc, and cadmium resistance, stress response associated with cold shock, osmotic and oxidative stress, and several phage-associated traits.

### Nucleotide sequence accession number.

The genome sequence of “*Candidatus* Cronobacter colletis” NCTC 14934^T^ has been deposited in GenBank under the accession no. JMSQ00000000.
